# Anti-IL-4Ralpha monoclonal antibody dupilumab mimics ulcerative colitis: a case report

**DOI:** 10.1186/s12876-021-01803-8

**Published:** 2021-05-08

**Authors:** Yosuke Shimodaira, So Takahashi, Katsunori Iijima

**Affiliations:** Department of Gastroenterology and Neurology, Akita University Graduate School of Medicine, 1-1-1 Hondo, Akita City, Akita 0108543 Japan

**Keywords:** Dupilumab, IL-4Ralpha, Ulcerative colitis

## Abstract

**Background:**

Various molecular-targeted therapeutic agents that inhibit cytokines and immune checkpoints are used in clinical practice. Some of these biologics that control immunity, such as anti-interleukin-17, anti-programmed cell death protein-1, and anti-cytotoxic T-lymphocyte-associated protein antibodies, affect intestinal immune homeostasis and cause intestinal inflammation. Development of enteritis due to dupilumab (an anti-IL-4Ralpha monoclonal antibody) therapy is not yet reported in the literature.

**Case presentation:**

A 17-year-old man was administered an injection of dupilumab and continued to receive it for refractory atopic dermatitis. After 3 months of initiating dupilumab therapy, he developed intermittent abdominal pain, tenesmus, and had diarrhea. Colonoscopy examination showed decreased vascularity, mild friability, and erythema in the cecum, part of the ascending colon, sigmoid colon, and rectum without any pathogenic bacteria. Histological examination revealed moderate mixed inflammatory cell infiltration, cryptitis, destruction of the crypt, decreased goblet cells, mucosal erosions, and edema. He was diagnosed with UC and was prescribed oral mesalazine (4800 mg/day) treatment. Within a month of the treatment, his diarrhea improved and the frequency of defecation decreased.

**Conclusions:**

This is a first report that dupilumab mimicked ulcerative colitis. Careful monitoring for adverse effects with the onset of an intestinal inflammation will be recommended after dupilumab administration.

## Background

Molecular-targeted therapeutic agents are innovative therapeutic agents used in several fields, such as cancer and immune diseases, that effectively act on specific molecules and in turn inhibit disease pathways [1, 2]. Various therapeutic agents that inhibit cytokines and immune checkpoints are used in clinical practice. However, some of these biologics that control immunity, such as anti-interleukin (IL)-17, anti-programmed cell death protein-1, and anti-cytotoxic T-lymphocyte-associated protein antibodies, affect intestinal immune homeostasis and mimic ulcerative colitis (UC) [3–5].

Intestinal immunity is mediated through cell signaling, and the immune system is tightly regulated even when exposed to foreign substances, such as intestinal microorganisms and dietary antigens. UC is a chronic intestinal inflammation caused by intestinal immune response dysregulation. It has been reported that T_H_2 cytokines are predominant in the intestinal mucosa in UC [6]; however, no amelioration of UC was observed after blocking the expression of T_H_2 cytokines in clinical trials [7, 8]. In addition, the development of enteritis due to dupilumab (an anti-IL-4Ralpha monoclonal antibody) therapy is not yet well understood.

An anti-IL-12/23p40 antibody has been used clinically as a cytokine-targeted therapy for UC [9]. However, it has been pointed out that while IL-23 regulates the differentiation function of T_H_17, and the inhibition of IL-17 conversely produces an inflammatory state that mimics UC [4]. Thus, the regulation of intestinal immunity in UC is complex and remains to be elucidated.

Here, we report a case in which dupilumab, an anti-IL-4Ralpha monoclonal antibody, mimics UC, an inflammatory bowel disease.

## Case presentation

**Fig. 1:**
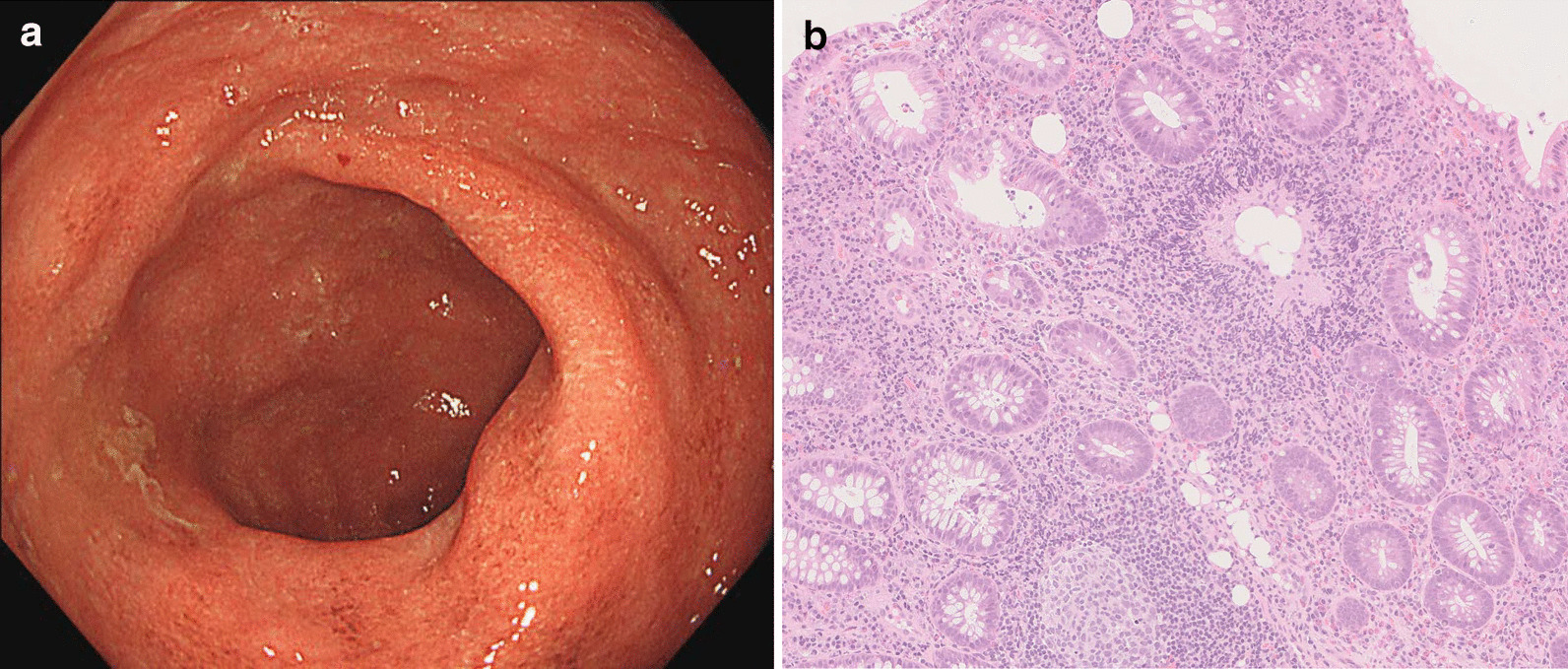
**a** Endoscopic image of rectum is shown. **b** Mucosal tissue was biopsied in sigmoid colon and histological examination with hematoxylin and eosin staining is shown with a scale bar

A 17-year-old man with a height of 168 cm and a weight of 70 kg presented to our dermatology department for the treatment of atopic dermatitis. He had a history of pediatric asthma and attention-deficit hyperactivity disorder and also allergies to house dust and pollen. Furthermore, his father had a medical history of UC. He had been treated with topical and oral therapy for refractory atopic dermatitis at a local clinic. Due to his refractory condition, he was administered an injection of dupilumab 600 mg and continued to receive it 300 mg every two weeks without any adverse events. After the administration of three doses, the skin rash on his back and trunk had almost disappeared. However, after 3 months of initiating dupilumab therapy, he developed intermittent abdominal pain, tenesmus, and had diarrhea seven times a day. Therefore, he was treated with polycarbophil calcium and lactomin for suspected irritable bowel syndrome but did not show any signs of improvement. He was referred to our department and underwent colonoscopy. The examination showed decreased vascularity, mild friability, and erythema in the cecum, part of the ascending colon, sigmoid colon, and rectum (Fig. 1a). No pathogenic bacteria were identified in the stool culture, and *Clostridioides difficile* toxin was also not detected. Blood tests showed no elevation of white blood cells, C reactive protein, or erythrocyte sedimentation rate, and there were no signs of anemia. Histological examination revealed moderate mixed inflammatory cell infiltration, cryptitis, destruction of the crypt, decreased goblet cells, mucosal erosions, and edema (Fig. 1b). Based on these findings, he was diagnosed with UC and was prescribed oral mesalazine (4800 mg/day) treatment. Within a month of the treatment, his diarrhea improved and the frequency of defecation decreased to three times a day. His atopic dermatitis continued to improve and dupilumab therapy was continued without any interruption for 1 year.

## Discussion and conclusions

We encountered a case where the administration of an IL-4Ralpha monoclonal antibody resulted in an inflammatory state mimicking UC. The endoscopic and histopathological findings closely resembled those of UC, leading to a UC diagnosis. The medical history of UC of his father suggested that he might have been genetically predisposed to develop UC, and it was assumed that chronic inflammation had developed due to inflammation triggers that caused immunological changes.

There have been various reports on the involvement of cytokines in UC. Many reports on effector cytokines have indicated that UC is a T_H_2 dominant disease [6, 10]. Dupilumab inhibits IL-4 and IL-13 signaling through IL-4Ralpha/IL-4RgammaC receptor dimer and IL-4Ralpha/IL-13Ralpha1 receptor dimer respectively and has been indicated for T_H_2-mediated allergic diseases with type 2 inflammation, such as atopic dermatitis and bronchial asthma [11]. There are two IL-13 receptor subtypes, IL-13Ralpha1 that forms a dimer with IL-4Ralpha, whereas IL-13Ralpha2 does not [12]. Therefore, dupilumab does not block IL-13Ralpha2 signaling. Although the function of IL-13Ralpha2 is yet unclear, the mucosal expression of IL-13Ralpha2 is found to affect the treatment of Crohn’s disease, which is another type of chronic inflammatory bowel disease [13]. It can be hypothesized that IL-13Ralpha2-mediated signaling plays an important role in the manifestation of an inflammatory state mimicking UC in this case. A previous study has reported the involvement of IL-17 in the development of UC, and the blocking of IL-17 expression causes an inflammatory condition mimicking UC [4], although anti-IL-12/23p40 antibodies which affects T_H_17 maintenance and function improve ulcerative colitis [9]. More than 200 disease susceptibility genes have been identified in UC. Although the odds ratio was not large for each gene, environmental factors were thought to play a crucial role in their development. In individuals with a genetic predisposition resulting in an altered intestinal immunity, IL-17 antibodies may influence the development of chronic intestinal inflammation. Actually, dupilumab downregulated mRNA expression of IL-17 but not that of IL-12/23p40 in atopic dermatitis [14]. The molecular signaling pathway interaction between IL-4Ralpha inhibition and T_H_17 is still needed to elucidated.

In this case, dupilumab was administered to treat refractory atopic dermatitis. A previous report indicated the association between atopic dermatitis and UC [15], and the fact that they are both T_H_2-dominant inflammations. Both these diseases may have a common risk factor as barrier dysfunction is involved in their pathology. UC is characterized by marked infiltration of eosinophils; however, no specific allergens have been identified. An abnormal immune response to intestinal bacteria has been suggested to be one of the causes of UC.

Of note, four patients were reported to have developed UC as an adverse event as per the post-marketing surveillance of dupilumab for bronchial asthma and atopic dermatitis conducted in Japan [16]. However, there have been no detailed reports on the onset of UC due to IL-4Ralpha antibodies. Although this case was mild, the development of an inflammatory state mimicking UC after administration of dupilumab was worthy of being reported. On this basis, careful monitoring for adverse effects is recommended after dupilumab administration so as the onset of an inflammatory state mimicking UC.

## Data Availability

Not applicable.

## Abbreviations

UC: Ulcerative colitis
IL: Interleukin
